# Histidine residues at the copper-binding site in human tyrosinase are essential for its catalytic activities

**DOI:** 10.1080/14756366.2020.1740691

**Published:** 2020-03-17

**Authors:** Hyangsoon Noh, Sung Jun Lee, Hyun-Joo Jo, Hye Won Choi, Sungguan Hong, Kwang-Hoon Kong

**Affiliations:** Department of Chemistry, College of Natural Sciences, Chung-Ang University, Seoul, South Korea

**Keywords:** Human tyrosinase, histidine residues, active site, CuA and CuB binding site, site-directed mutagenesis

## Abstract

Tyrosinase is a copper-binding enzyme involved in melanin biosynthesis. However, the detailed structure of human tyrosinase has not yet been solved, along with the identification of the key sites responsible for its catalytic activity. We used site-directed mutagenesis to identify the residues critical for the copper binding of human tyrosinase. Seven histidine mutants in the two copper-binding sites were generated, and catalytic activities were characterised. The tyrosine hydroxylase activities of the CuA site mutants were approximately 50% lower than those of the wild-type tyrosinase, while the dopa oxidation activities of the mutants were not significantly different from that of wild-type tyrosinase. By contrast, mutations at CuB significantly decreased both tyrosine hydroxylation and dopa oxidation activities, confirming that the catalytic sites for these two activities are at least partially distinct. These findings provide a useful resource for further structural determination and development of tyrosinase inhibitors in the cosmetic and pharmaceutical industries.

## 1. Introduction

Melanin biosynthesis is a complicated pathway involving chemical and enzymatic reactions and is limited to melanocytes in mammals. Tyrosinase (monophenol monooxygenase, EC 1.14.18.1) plays a pivotal role in the melanin synthesis pathway. Moreover, tyrosinase is the only human melanogenic enzyme with well-established *in vivo* catalytic enzyme activity^1^, catalysing several steps in melanin synthesis and generated by the hydroxylation of l-tyrosine^2–4^. Tyrosinase is a copper-containing metalloprotein belonging to the type-3 copper protein family, together with haemocyanins and catechol oxidases. These proteins are abundant in mammals, bacteria, fungi, and plants, and the active sites are highly conserved among the different species^5^. By synthesising melanin, tyrosinase exerts a protective function in UV-induced damage^6^ but can also cause hyperpigmentation, leading to aesthetic problems and melanoma. Moreover, the lack of tyrosinase activity is associated with oculocutaneous albinism (OCA) in many animal species, including humans^7^^,^^8^. As such, human tyrosinase is a quite attractive target for medical and industrial applications. Particularly, the screening of potent antagonists of tyrosinase and their subsequent development to drugs have attracted substantial interest in the cosmetic industry.

To date, two crystal structures of catechol oxidase^9^^,^^10^, three crystal structures of haemocyanin^11–13^, and three crystal structures of tyrosinase – from *Agaricus bisporus*^14^, *Streptomyces castaneoglobisporus*^15^, and *Bacillus megaterium*^16^ – have been resolved. Unfortunately, there is still no crystal structure of human tyrosinase; however, a reliable model could be generated based on the amino acid sequence and previously reported active sites^17^. The mature human tyrosinase consists of 529 amino acids including a short *N*-terminal signal peptide targeting the nascent polypeptide to the endoplasmic reticulum for folding, sorting, and, modification^18^. Further, it contains seven *N*-glycosylation motifs, two putative copper-coordinating sites distinct to CuA and CuB, one transmembrane domain, and a short carboxyl tail that has the important signals for targeting and sorting to melanosomes^19^. Moreover, six histidine residues in the active site are involved in coordination with the two copper ions (CuA and CuB)^20^. However, there is no clear evidence for the direct binding of human tyrosinase to copper. The alignment of amino acid sequence for tyrosinases, catechol oxidases, and haemocyanins suggested that the two homologous regions distinct to CuA and CuB could be directly involved in the two copper bindings, which are critical for the catalytic activities of the enzymes^21^. Furthermore, a mutational study of human tyrosinase revealed that both copper binding sites are necessary for the catalytic activity of the enzyme and for copper binding at the active site^22^.

**Figure 1.**
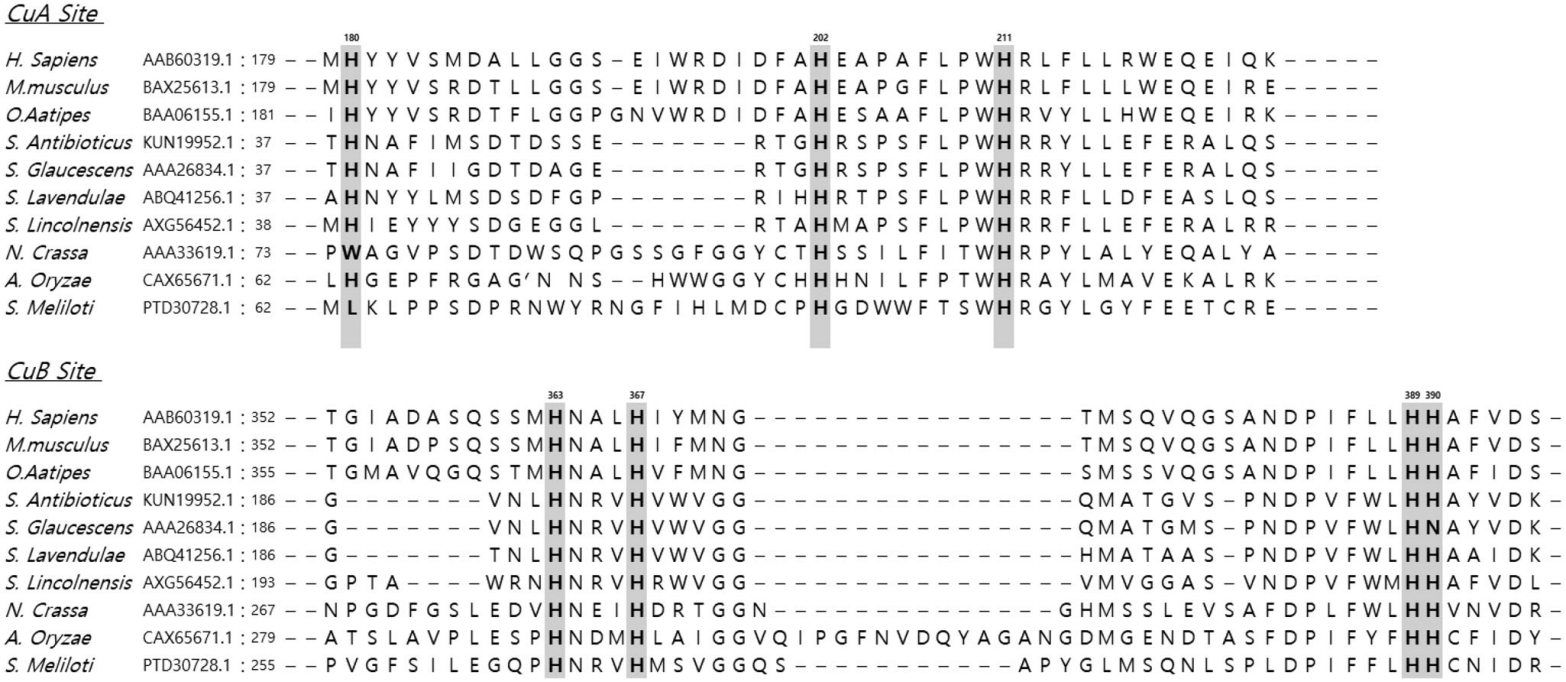
Amino acid sequence alignment of tyrosinases from *Homo sapiens*, *Mus musculus, Oryzias latipes, Streptomyces antibioticus, Streptomyces glaucescens, Streptomyces lavendulae, Streptomyces lincolnensis, Neurospora crassa, Aspergillus oryzae,* and *Sinorhizobium meliloti.* The numbers correspond to human tyrosinase amino acid positions, and copper ligands in human tyrosinase are indicated.

In this study, we sought to demonstrate whether the seven copper-binding histidine residues (H180, H202, H211, H363, H367, H389 and H390) around the two copper binding sites are necessary for the catalytic activities and folding of tyrosinases. Nakamura et al.^23^ reported that seven histidine residues – H63, H84, H93, H290, H294, H332, and H333 – are essential for the tyrosinase activity of *Aspergillus oryzae* and demonstrated that replacement of each residue with asparagine abolished the catalytic activities of the mutant enzymes. Moreover, a crystallographic analysis of *Palinurus interruptus* haemocyanin showed that one of the pairs of copper ions, CuA, is enclosed by residues H196, H200 and H226, while the other, CuB, is surrounded by residues H346, H350 and H386^12^. Against this background, in the present study, the mutation positions were selected by the amino acid sequence alignment of *H. sapiens* tyrosinase with those of other tyrosinases from *Mus musculus, Oryzias latipes, Streptomyces antibioticus, Streptomyces glaucescens, Streptomyces lavendulae, Streptomyces lincolnensis, Neurospora crassa, A. oryzae* and *Sinorhizobium meliloti* (Figure 1), focussing on the positions of seven histidine residues (H180, H202, H211, H363, H367, H389 and H390). Further, we replaced each histidine residue around the CuA and CuB sites with a non-polar amino acid, alanine, using a site-directed mutagenesis approach. We then compared the hydroxylation and oxidation activities of these mutants. These findings shed light on the essential residues responsible for the catalytic activity of human tyrosinase, which would be of enormous value in predicting the structure and designing new inhibitors.

## 2. Materials and methods

### 2.1. Cloning and site-directed mutagenesis of human tyrosinase

We previously reported the cloning of the human tyrosinase gene into the pHis vector, along with protein expression and purification^24^. To improve these last two steps, in this study, we used a PCR approach to reclone the gene into the pET-26b(+) bacterial vector (Novagen, Madison, WI, USA), which contains a 6× His-tag at the *C*-terminal, using *Nde* I and *Xho* I (Takara Shuzo, Otsu, Shiga, Japan) as restriction sites, including the stop codon.

Site-directed mutagenesis to substitute the six histidine residues with alanine was carried out using QuikChange Site-Directed Mutagenesis Kit (Agilent, #200518) according to the manufacturer’s manual.

Primers (COSMO Genetech, Seoul, South Korea) used for cloning and mutation generation are listed in Table 1. Each plasmid for the wild type and mutants was transformed into XL1-blue competent cells and sequenced (Bionics, Seoul, South Korea). Plasmids confirmed by sequencing were then transformed into the *Escherichia coli* strain BL21 Star (DE3) (Novagene) for enzyme expression.

**Table 1.** Primers used for recloning into pET26b(+) and site-directed mutagenesis.

Enzymes	Sequence of primers^a^	Direction
Wild type	5′-GGAATTCCATATGCACTTCCCTAGAGCCTGTGTCTCCTCT-3′	Forward
	5′-ATCCGCTCGAGCGGTAAATGGCTCTGATACAAGCTGTG-3′	Reverse
H180A	5′-CTTTGTCTGGATGGCTTATTATGTGTCAA-3′	Forward
	5′-TTGACACATAATAAGCCATCCAGACAAAG-3′	Reverse
H202A	5′-CATTGATTTTGCCGCTGAAGCACCAGCTT-3′	Forward
	5′-AAGCTGGTGCTTCAGCGGCAAAATCAATG-3′	Reverse
H211A	5′-TTTTCTGCCTTGGGCTAGACTCTTCTTGT-3′	Forward
	5′-ACAAGAAGAGTCTAGCCCAAGGCAGAAAA-3′	Reverse
H363A	5′-TCTCAAAGCAGCATGGCCAATGCCTTGCACATC-3′	Forward
	5′-GATGTGCAAGGCATTGGCCATGCTGCTTTGAGA-3′	Reverse
H367A	5′-ATGCACAATGCCTTGGCCATCTATATGAATGGA-3′	Forward
	5′-TCCATTCATATAGATGGCCAAGGCATTGTGCAT-3′	Reverse
H389A	5′-CCTATCTTCCTTCTTGCCCATGCATTTGTTGAC-3′	Forward
	5′-GTCAACAAATGCATGGGCAAGAAGGAAGATAGG-3′	Reverse
H390A	5′-ATCTTCCTTCTTCACGCTGCATTTGTTGACAGT-3′	Forward
	5′-ACTGTCAACAAATGCAGCGTGAAGAAGGAAGAT-3′	Reverse

^a^ Changed bases are underlined.

### 2.2. Recombinant human tyrosinase expression in E. coli

To express recombinant wild-type and mutant tyrosinase, BL21 Star (DE3) transformed with each construct was inoculated into Luria-Bertani (LB) culture medium supplemented with 30 μg/mL kanamycin (Sigma-Aldrich, St. Louis, MO, USA) and 1 mM CuSO_4_, and then induced with 0.4 mM isopropyl-β-d-thiogalactopyranoside (IPTG; Sigma-Aldrich) at an optical density 600 nm of 0.3–0.4 for 12 h. Centrifugation was carried out at 20,000*g* for 30 min at 4 °C to harvest the cells, and collected cells were washed three times with 50 mM Tris–HCl buffer with 1% Triton X-100 (Buffer A, pH 6.8), and resuspended in 10 ml of Buffer A containing 1 mM CuSO_4_, 5 mM EDTA, and 100 μM PMSF. Resuspended cells were then lysed in a sonicator (Qsonica, Newtown, CT, USA) for 20 min at 30–40 W with 9-s pulse on and 1-s pulse off. After centrifuging the lysate at 20,000*g* for 30 min at 4 °C, the supernatant was collected and stored at 4 °C until analysis.

### 2.3. Recombinant human tyrosinase purification

The His-tagged wild-type tyrosinase and mutant enzymes were purified by loading the lysate on a diethylaminoethyl (DEAE)-Sephacel column, following immobilisation in a metal-affinity column (Pharmacia Biotech, Uppsala, Sweden). The unbound protein fractions after passing through the DEAE-Sephacel column were applied to a metal affinity column with Ni-NTA resin (Novagen). The column was rinsed with 50 mM Tris–HCl buffer containing 500 mM NaCl, 1% Triton X-100 (Buffer B, pH 7.8), and 20 mM imidazole. The elution procedure was performed with Buffer B containing 150 mM imidazole. The imidazole in the collected proteins was removed by dialysis with Buffer A, and the purified wild-type and mutant enzymes in Buffer A were then used for subsequent experiments.

### 2.4. Enzyme activity assay and kinetics analysis

#### 2.4.1. Tyrosine hydroxylase activity assay

The tyrosine hydroxylase activity of wild-type and mutant human tyrosinase was determined as described previously^25^^,^^26^. The reaction mixtures containing 100 μM l-tyrosine, 5 μM l-3,4-dihydroxyphenylalanine (l-dopa, Sigma-Aldrich), 4 mM ascorbic acid, 0.5 μM CuSO_4_, and tyrosinase enzyme in a total volume of 2.5 ml were incubated at 37 °C for 3 h. The l-dopa generation in the reaction was measured by fluorescence at 360 nm excitation and 490 nm emission, and the tyrosine hydroxylase activity was determined. The specific activities for tyrosine hydroxylase were determined as 1 mmol mM of l-dopa produced per hour per microgram of protein under the above reaction conditions. The reaction rates without enzyme or l-tyrosine served as negative controls and were used as a normalisation factor for each enzyme reaction rate.

#### 2.4.2. Dopa oxidase activity assay

The dopa oxidase activity of tyrosinase was measured by spectrophotometric monitoring of dopachrome production at 475 nm as described previously^4^. The reaction mixtures containing 50 mM Tris–HCl buffer (pH 7.5), 3 mM l-dopa (ε_dopachrome_ = 3600 M^−1 ^cm^−1^), 1 mM CuSO_4_, and 50 µL of enzyme in a total volume of 1 ml were incubated at 37 °C for 3 min. The unit of dopa oxidase activity was determined as 1 μmol of dopachrom production per minute of the reaction. The specific activities of dopa oxidase were determined by units of dopa oxidase activity per milligram of enzyme. The steady-state rates were calculated from the linear slope of time versus absorbance curves. The reaction rates without enzyme or l-dopa served as negative controls and were used as a normalisation factor for each enzyme reaction rate.

#### 2.4.3. Enzyme kinetics analysis

The *K*_m_ and *k*_cat_ values for l-tyrosine and l-dopa were calculated by the Lineweaver–Burk plot^27^ with five separate experiments. The concentration of purified wild-type and mutant proteins was measured by the bicinchoninic acid (BCA) assay (Thermo Fisher, Waltham, MA, USA) using bovine serum albumin (BSA) as a standard protein.

### 2.5. Electrophoresis

Denatured wild-type and mutant proteins (20 μg) were loaded onto 12% sodium dodecyl sulphate-polyacrylamide (SDS-PAGE) gels. After separation by electrophoresis, Coomassie brilliant blue R-250 was used to stain the gel, and the protein bands were visualised with 40% methanol and 10% glacial acetic acid.

### 2.6. Three-dimensional structure modelling of tyrosinase

Structural modelling of tyrosinase was performed by ‘SWISS-MODEL EXPASY’ software provided by Biozentrum, the Centre for Molecular Life Sciences in Basel University (Basel, France). Modulation of predicted protein models was carried out with ‘Autodock tools’ software provided by the Scripps Research Institute (CA, USA). Visualisation of protein models was performed by ‘UCSF Chimaera package’ from the Resource for Biocomputing, Visualisation and Informatics (RBVI) of the University of California (CA, USA)

### 2.7. Statistical analysis

Results are expressed as mean ± standard deviation. Data were analysed using an unpaired two-tailed Student *t*-test to detect the significance of differences between groups. *p* < 0.05 was considered statistically significant.

## 3. Results

### 3.1. Expression and purification of the recombinant wild-type and mutant tyrosinase

**Figure 2.**
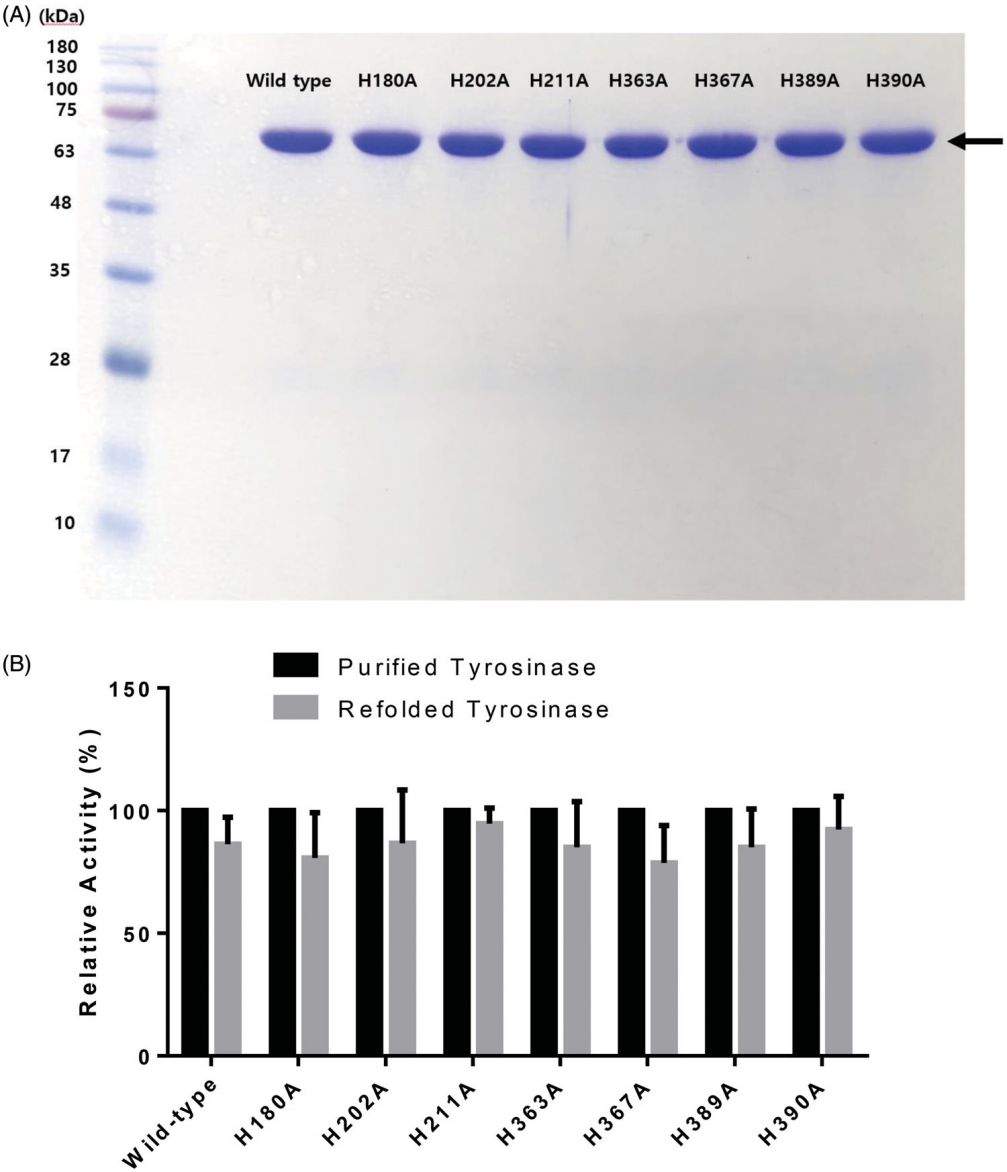
(A) SDS-PAGE of wild-type human tyrosinase and mutant enzymes after purification through diethylaminoethyl (DEAE)-Sephacel and immobilised metal-affinity chromatography. The gel was stained with Coomassie blue. The arrow indicates the calculated size of 66 kDa, corresponding to human tyrosinase. (B) Confirmation of tyrosinase stability by refolding. The tyrosinase expressed and purified in *E. coli* BL21 was denatured by adding 8 M urea, and then refolded by gradationally reducing the urea concentration by dialysis. The activity of the refolded tyrosinase was measured and compared to that of the early purified tyrosinase. The values represent the mean ± SD (*n* = 3).

Compared with our previous pHis-Tyrosinase recombinant bacterial plasmid that contains the human tyrosinase sequence and an *N*-terminal poly-histidine tag^24^, recloning of the tyrosinase gene into the pET26b(+) expression plasmid containing a *C*-terminal 6x His-tag successfully enhanced enzyme expression and catalytic activity. The recombinant pET26b(+)- human tyrosinase (pET-hTyr) could produce large amounts of pure and active tyrosinase (66 kDa) in the bacterial expression system. A high yield (approximately 34%, data not shown) of tyrosinase expression was obtained using *E. coli* BL21 Star (DE3) cells followed by purification, which was greater than that obtained in our previous study (approximately 19%)^24^, demonstrating the efficiency of the 6× His tag at the *C*-terminal rather than the *N*-terminal. As a result, the final purification was determined to be enhanced by 49-fold, and approximately 2.75 mg/L of purified tyrosinase was obtained. The purified recombinant human tyrosinase exhibited a single protein band corresponding to a molecular weight of 66,000 on the SDS-PAGE gel (Figure 2), which was used for subsequent characterisation.

All of the purified tyrosinase mutants (H180, H202, H211, H363, H367, H389 and H390) were prepared under the same conditions, and appeared as single protein bands on the SDS-PAGE gel (Figure 2; Supplementary Table 1). All mutants showed acceptable soluble, expressed, and purified enzyme concentrations in comparison with the wild-type tyrosinase.

### 3.2. Stability of the recombinant wild-type tyrosinase and mutants

The stability of the purified wild-type tyrosinase and mutants was confirmed by refolding of proteins. The refolded wild-type tyrosinase exhibited similar activity to the wild-type tyrosinase expressed in *E. coli*, indicating that the tyrosinase expressed in BL21(DE3) has a stable protein structure (86.3% vs. control, *p* = 0.0981, Figure 2(B)). Furthermore, all of the purified tyrosinase mutants showed similar activities between purified and refolded proteins (purified vs. refolded, *p* > 0.05 for all mutants, Figure 2(B)).

### 3.3. Catalytic activities of the recombinant wild-type tyrosinase and mutants

**Figure 3.**
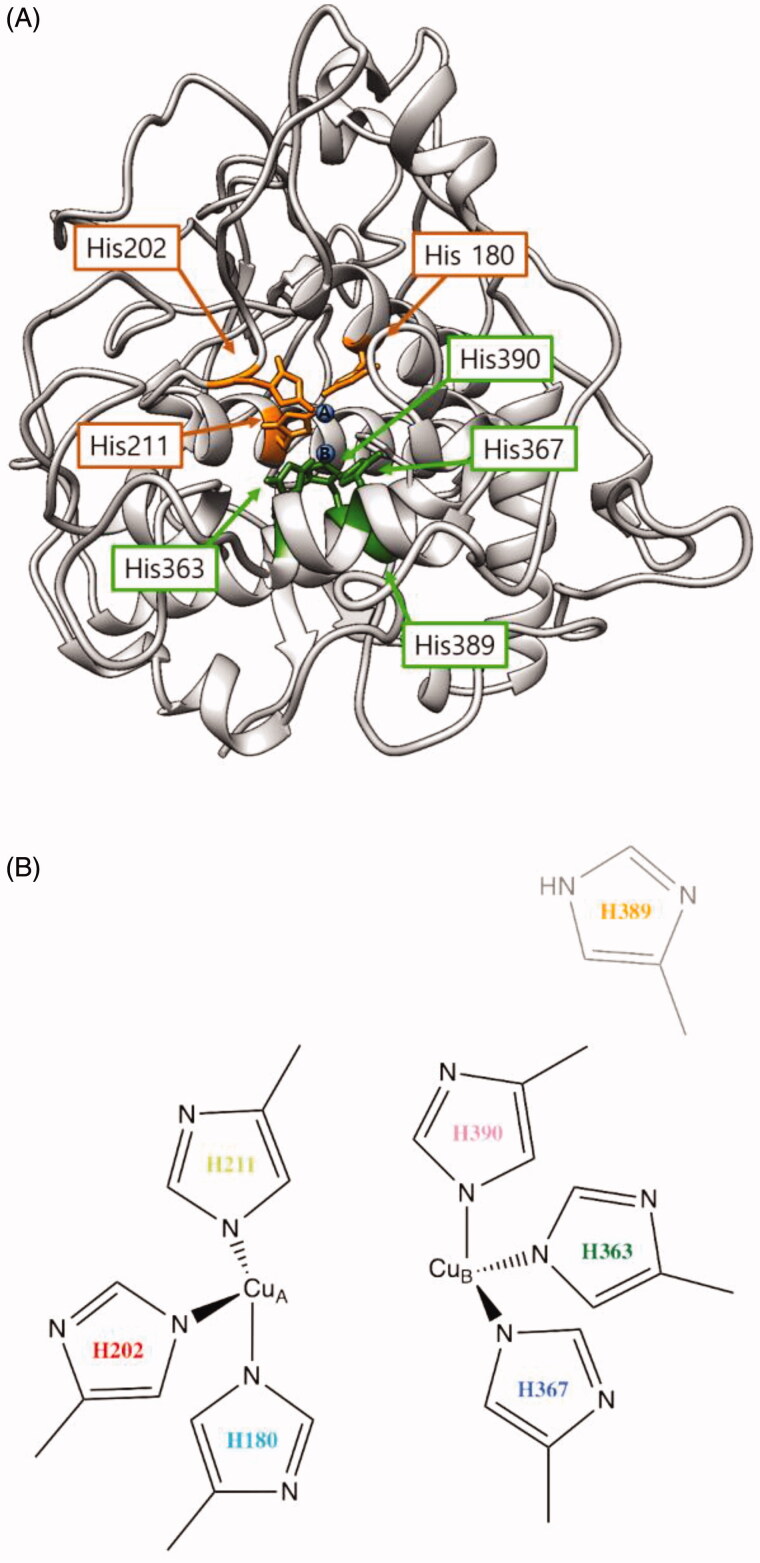
(A) Proposed three-dimensional structure of human tyrosinase. The di-copper site was found in the centre of the four-helix bundle motif. H180, H202 and H211 residues show direct binding with the copper atom at the CuA site, and H363, H367 and H390 are directly coordinated with the copper atom at the CuB site. However, H389 is located around the CuB site without direct binding. (B) Schematic illustration of CuA- and CuB-binding sites of human tyrosinase. Based on three-dimensional structure modelling, the residues H180, H202, and H211 show direct binding with CuA, whereas H363, H367 and H390 are directly coordinated with CuB. H389 is located around CuB but without direct binding.

The wild-type tyrosinase was expressed and purified as a functional enzyme, showing clear hydroxylation and oxidation activities, and all six mutants showed lower activities than those of the wild-type (Table 2). The majority of the mutant enzymes, except for the H389A mutant, showed significantly lower tyrosine hydroxylase (monophenolase) activity for the l-tyrosine substrate, suggesting that these substituted residues are required for tyrosine hydroxylation activity, whereas dopa oxidase (diphenolase) activity was reduced significantly in four mutants (H363A, H367A, H389A and H390A) around the CuB site. Three mutants (H180A, H202A and H211A) around the CuA site retained partial activity up to 88%, 76% and 89%, respectively, for the dopa oxidation reaction compared with that of wild-type tyrosinase. This finding suggested that the copper binding of the three histidine residues around CuA is essential for binding to the aromatic substrate, l-tyrosine. Moreover, structure modelling for human tyrosinase using Swiss-model Expasy software based on tyrosinase-related protein 1 structure – which is already resolved with X-ray crystallography^28^ – predicted that these three residues are also involved in direct binding to the copper atom (Figure 3).

**Table 2.** Specific activity of the wild-type tyrosinase and mutant enzymes for l-tyrosine hydroxylation and l-dopa oxidation reactions.

Enzyme	l-Tyrosine		l-Dopa	
	Specific activity (μmol·μg^−1^·h^−1^)	Relative activity (%)	Specific activity (units/mg)	Relative activity (%)
Wild type	108.3 ± 2.1	100	1.53 ± 0.10	100
H180A	47.2 ± 1.2	44	1.35 ± 0.02	88
H202A	43.5 ± 0.9	40	1.17 ± 0.01	76
H211A	51.6 ± 0.8	48	1.37 ± 0.03	89
H363A	24.9 ± 0.3	23	0.23 ± 0.02	15
H367A	20.6 ± 0.3	19	0.34 ± 0.03	22
H389A	110.5 ± 0.2	102	1.04 ± 0.03	68
H390A	17.3 ± 0.5	16	0.40 ± 0.05	26

Values are means ± SD, generally based on *n* ≥ 5.

### 3.4. Kinetic analysis of the recombinant wild-type and mutant tyrosinase

Kinetic properties of the wild-type and mutant tyrosinase were obtained by the Lineweaver–Burk plot and are shown in Table 3. The *K*_m_ values of the mutant enzymes for the l-tyrosine hydroxylation reaction showed no significant difference compared with those of the wild-type enzyme. This result indicated that histidine residues around both the CuA and CuB sites are not involved in l-tyrosine substrate binding. By contrast, the *K*_m_ values of the mutant enzymes for the l-dopa oxidation reaction were more variable. The three mutants around the CuA site showed *K*_m_ values similar to those of the wild-type enzyme, but *K*_m_ values of the H363A and H367A mutants were nine-times lower than those of the wild-type enzyme, suggesting that these two histidine residues may affect the binding affinity with the l-dopa substrate as well as copper binding. Furthermore, according to the *k*_cat_ values for the wild-type tyrosinase and mutants, the three histidine residues bound to the CuA site are more critical for the l-tyrosine hydroxylation reaction, whereas the histidine residues at the CuB site play a more essential role for the l-dopa oxidation reaction (Table 3).

**Table 3.** Kinetic parameters of the wild-type tyrosinase and mutant enzymes for l-tyrosine hydroxylation and l-dopa oxidation reactions.

Enzyme	l-Tyrosine			l-Dopa		
	*K*_m_ (μM)	*k*_cat_ (s^−1^)	*k*_cat_/*K*_m_ (μM^−1^ s^−1^)	*K*_m_ (mM)	*k*_cat_ (s^−1^)	*k*_cat_/*K*_m_ (mM^−1^ s^−1^)
Wild type	1.32 ± 0.12	0.61 ± 0.025	0.46	0.34 ± 0.08	34.1 ± 2.3	100.3
H180A	1.81 ± 0.12	0.34 ± 0.012	0.19	0.42 ± 0.07	28.5 ± 1.9	67.9
H202A	1.63 ± 0.11	0.42 ± 0.017	0.26	0.45 ± 0.07	30.0 ± 2.5	66.7
H211A	1.44 ± 0.07	0.50 ± 0.043	0.34	0.37 ± 0.09	32.4 ± 3.1	87.6
H363A	1.99 ± 0.08	0.44 ± 0.01	0.22	3.09 ± 0.12	17.2 ± 1.3	5.6
H367A	1.77 ± 0.15	0.48 ± 0.011	0.27	3.20 ± 0.09	15.8 ± 1.0	4.9
H389A	1.67 ± 0.10	0.63 ± 0.018	0.38	0.24 ± 0.05	38.4 ± 1.2	160.0
H390A	1.47 ± 0.13	0.52 ± 0.017	0.35	0.36 ± 0.07	29.7 ± 1.3	82.5

Values are means ± SD, generally based on *n* ≥ 5.

In the predicted 3 D tyrosinase model (Figure 3), the most likely binding sites of tyrosinase for CuA were determined to be H180, H202 and H211, which directly bind to copper atoms. Similarly, at the CuB site, the copper atom was coordinated by three histidine residues, H363, H367 and H390, through direct binding. H389 could be involved in catalytic reactions with indirect cooperation at the CuB region in human tyrosinase.

## 4. Discussion

In this study, recombinant wild-type and mutant human tyrosinase enzymes were overexpressed in *E. coli* BL21 (DE3) and utilised to analyse the catalytic activity. It has been reported that *N*-glycosylation is necessary for human tyrosinase stability and activity *in vivo*^29–31^. However, our previous studies and those of another group have also shown that recombinant human tyrosinase from *E. coli* could exhibit activity without phosphorylation or glycosylation^18^^,^^24^^,^^32^. As expected, we could detect the catalytic activity of recombinant human tyrosinase expressed in BL21 (DE3) in this study.

Previous studies of tyrosinase suggested that histidine residues are critical for its catalytic activity. In *A. oryzae* tyrosinase, the replacement of three histidine residues (H63, H84 and H93) with asparagine at the CuA binding site largely abolished copper binding by approximately 50%, revealing that the histidine residues are required for copper binding. Moreover, these residues were deemed to be critical for catalysis, given the diminishment of the copper content and the abolishment of tyrosine hydroxylase and dopa oxidase activities with l-tyrosine and l-dopa, respectively^23^. Similarly, copper contents in the histidine residue substituents (H93A, H116A and H125A) of a polyphenol oxidase, type-3 copper enzyme of *C. grandiflora*, were decreased, confirming that the histidine residues are critical for copper binding^33^. Consistent with these previous reports, in this study, we confirmed that three histidine residues, H180, H202 and H211, of human tyrosinase around the CuA site are essential for enzyme activity as determined by site-directed mutagenesis. These three histidine residues may be directly involved in copper binding to catalyse the l-tyrosine hydroxylation reaction.

H290, H284, H332 and H333 around the CuB site were found to be required for catalytic function and to act as copper-coordinating ligands of the mature tyrosinase from *A. oryzae*^23^. Moreover, Spritz et al.^22^ described that human tyrosinase contains four conserved histidine residues (H363, H367, H389 and H390). They further showed that the H390A mutant eliminated catalytic activity, but did not reduce copper binding, whereas the H389 replacement resulted in complete lack of activity. This suggested that only H389 plays an essential role in copper binding of human tyrosinase, despite the recognition of H390 as a conserved residue in all putative copper binding sites^22^. However, we found that the H363, H367 and H390 residues of human tyrosinase are likely directly involved in copper binding based on evaluations of catalytic activity and structural modelling. Furthermore, in contrast to these previous findings, H389 did not abolish the catalytic activity for l-tyrosine hydroxylation and showed indirect binding with the copper atom at the CuB site. Although we showed that H389 was indirectly involved in tyrosine hydroxylation reaction, its precise role at the CuB site requires further investigation.

Although the CuB site exhibits a higher conservation than CuA, studies of tyrosinase from different species have shown the involvement of the conserved histidine residues in copper binding. The conserved histidine residues around copper ions may play different roles in stability, flexibility, and additional structure conformation for the catalytic activity of tyrosinase. According to structure studies in other species, H54 in tyrosinase from *S. castaneoglobisporus* (TyrSc) was ligated to CuA that is transferred from the caddie protein through H82, M84 and H97 to the flexible H54 residue, which changes its conformation to coordinate the copper in the active site^34^. In *B. megaterium* tyrosinase (TyrBm), M61 and M184 residues transfer copper ions to the flexible H60 (in the same manner as H54 in TyrSc), which positions the copper ion in the active site^35^. Furthermore, H251 residues of *A. bisporus* tyrosinase coordinate CuB in the active site, which consequently causes CuB flexibility in the active site^36^. A structural study of tyrosinase from *A. oryzae* (TyrAo) showed that the thioether bond between H94 and C92 is formed only in the presence of copper in the active site^37^.

Moreover, it has been proposed that the proton of the hydroxyl group of monophenols may be removed and transferred to one of the coordinating histidine residues in the catalytic mechanism of tyrosinase^38^^,^^39^. The deprotonated substrates are bound to the active centre of tyrosinase^40^, and this may be stabilised by the interaction with the conserved histidine residues serving as a proton acceptor, even though this should be proved by compelling evidence.

Furthermore, recent studies have identified that an Asn residue along with a Glu residue is critical for tyrosinase tyrosine hydroxylation activity at Cu binding sites, showing that polyphenol oxidase, which lacks monophenolase activity, can be transformed to a tyrosinase by simply introducing an asparagine. A conserved water molecule activated by asparagine and glutamate is proposed to mediate the deprotonation of the monophenol at the active site^41^^,^^42^. Another study showed that an alternative set of four key residues, including a conserved glutamate and phenylalanine, are located around copper active sites with conserved copper-coordinating histidine residues^43^. The present study thus further expands this mechanism by demonstrating the key role of histidine residues of tyrosinase for its catalytic activity, although further study may be needed to elucidate the role of each histidine residue in this process.

Thus, we conclude that one of a pair of copper ions, CuA, directly binds to three histidine residues (H180, H202 and H211), while the other, CuB, directly binds to three other histidine residues (H363, H367 and H390) and indirectly binds to H389. We further assume that copper coordination at one site may facilitate copper binding at the other site. However, the crystal structure of human tyrosinase is required to identify the crucial residues for copper binding in catalysing reactions.

## Supplementary Material

Supplemental Material
