# Supplementary material for: Histidine residues at the copper-binding site in human tyrosinase are essential for its catalytic activities
Source: J Enzyme Inhib Med Chem. 2020 Mar 17;35(1):726–32. doi: 10.1080/14756366.2020.1740691 (PMC7144311; doi:10.1080/14756366.2020.1740691)
Supplement: Supplemental Material [file IENZ_A_1740691_SM1460.pdf]

Supplementary Material

Supplementary Table 1. Purification of wild-type and mutant tyrosinase from *E. coli*.

Proteins	Steps	Total proteins (mg)	Total activity (units)	Specific activity (units/mg)	Yield (%)	Purification (fold)
WT	Cell lysate	400	12.8	0.032	100.0	1.0
	DEAE[a]	64	10.0	0.16	78.2	4.9
	IMAC[b]	2.75	4.21	1.531	32.9	47.8
H180A	Cell lysate	375	11.25	0.030	100.0	1.0
	DEAE[a]	62	8.52	0.137	75.7	4.6
	IMAC[b]	2.60	3.51	1.350	31.2	44.9
H202A	Cell lysate	382	9.71	0.025	100.0	1.0
	DEAE[a]	48	6.79	0.141	69.9	5.6
	IMAC[b]	2.66	3.12	1.173	32.1	46.1
H211A	Cell lysate	420	11.35	0.027	100.0	1.0
	DEAE[a]	65	8.21	0.126	72.3	4.7
	IMAC[b]	2.25	3.08	1.369	27.1	50.7
H363A	Cell lysate	365	1.92	0.005	100.0	1.0
	DEAE[a]	55	1.25	0.023	65.1	4.3
	IMAC[b]	2.52	0.58	0.230	30.2	43.8
H367A	Cell lysate	409	2.84	0.007	100.0	1.0
	DEAE[a]	63	1.98	0.031	69.7	4.5
	IMAC[b]	2.13	0.73	0.343	25.7	49.4
H389A	Cell lysate	384	8.68	0.023	100.0	1.0
	DEAE[a]	59	6.79	0.115	78.2	5.1
	IMAC[b]	2.87	2.99	1.042	34.4	46.1
H390A	Cell lysate	362	3.25	0.009	100.0	1.0
	DEAE[a]	59	2.52	0.043	77.5	4.8
	IMAC[b]	2.31	0.93	0.403	28.6	44.8

a Diethylaminoethyl-Sephacel chromatography.

b Immobilised metal affinity chromatography.

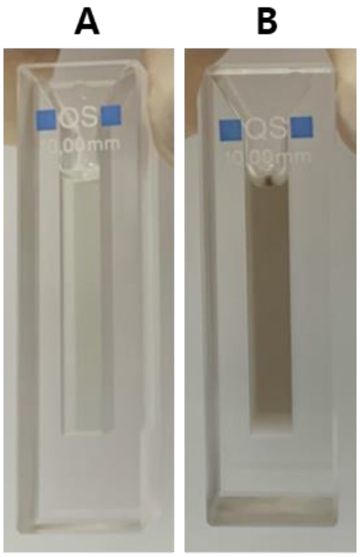

Supplementary Figure S1. Change in colour of the tyrosinase oxidation reaction. Representative image of the colour change of the tyrosinase oxidation reaction. A. Negative control reaction that did not include tyrosinase enzyme; B. Tyrosinase experimental reaction.
